# HCMV-Encoded NK Modulators: Lessons From *in vitro* and *in vivo* Genetic Variation

**DOI:** 10.3389/fimmu.2018.02214

**Published:** 2018-10-01

**Authors:** Mihil Patel, Virginia-Maria Vlahava, Simone K. Forbes, Ceri A. Fielding, Richard J. Stanton, Eddie C. Y. Wang

**Affiliations:** Division of Infection and Immunity, Cardiff University School of Medicine, Cardiff, United Kingdom

**Keywords:** HCMV, immune modulation, NK cells, NK evasion, genetic variation

## Abstract

Human cytomegalovirus (HCMV) is under constant selective pressure from the immune system *in vivo*. Study of HCMV genes that have been lost in the absence of, or genetically altered by, such selection can focus research toward findings of *in vivo* significance. We have been particularly interested in the most pronounced change in the highly passaged laboratory strains AD169 and Towne—the deletion of 13–15 kb of sequence (designated the U_L_/b′ region) that encodes up to 22 canonical genes, UL133-UL150. At least 5 genes have been identified in U_L_/b′ that inhibit NK cell function. UL135 suppresses formation of the immunological synapse (IS) by remodeling the actin cytoskeleton, thereby illustrating target cell cooperation in IS formation. UL141 inhibits expression of two activating ligands (CD155, CD112) for the activating receptor CD226 (DNAM-1), and two receptors (TRAIL-R1, R2) for the apoptosis-inducing TRAIL. UL142, ectopically expressed in isolation, and UL148A, target specific MICA allotypes that are ligands for NKG2D. UL148 impairs expression of CD58 (LFA-3), the co-stimulatory cell adhesion molecule for CD2 found on T and NK cells. Outside U_L_/b′, studies on natural variants have shown UL18 mutants change affinity for their inhibitory ligand LIR-1, while mutations in UL40's HLA-E binding peptide differentially drive NKG2C^+^ NK expansions. Research into HCMV genomic stability and its effect on NK function has provided important insights into virus:host interactions, but future studies will require consideration of genetic variability and the effect of genes expressed in the context of infection to fully understand their *in vivo* impact.

## Introduction

Human cytomegalovirus (HCMV) is a persistent infection that rarely causes serious clinical symptoms in healthy adults, but opportunistically induces life-threatening disease with long-term sequalae in the immune compromised such as transplant recipients, AIDS patients and following infection of the fetus. NK cells play a critical role in preventing HCMV disease, as evidenced by serious complications due to HCMV infection in individuals with NK cell deficiency (1, 2). This has driven extensive research into the interaction between NK cells and HCMV, revealing a steadily increasing number of viral-encoded immunomodulatory functions. However, relating *in vitro* function to *in vivo* significance remains difficult, not least due to the necessary caution in interpreting data from experiments using different HCMV strains, many of which do not encode a wildtype complement of viral genes (3).

Like many viruses, rapid selection of mutants can occur when HCMV is placed under selective pressure. *In vivo*, there is pressure to retain immunomodulatory functions due to selection from the host immune system, yet this is not the case *in vitro*. Only 26% (45 of 171) of the canonical HCMV genes are necessary for replication *in vitro* (4, 5), and selection of mutants lacking genes that are either not required, or are inhibitory, *in vitro*, occurs within a few weeks of isolation, becoming more extensive as passage continues (6–9). Ultimately numerous mutations can be seen, including large deletions such as the 13–15 kb U_L_/*b*′ region, UL133-UL150, that has been lost from the widely used AD169 and Towne strains (7, 10). Not only does this progressive loss of genetic material mean laboratories are forced to work with strains lacking many genes that are relevant to understanding the interaction of the virus with the host, but the “same” virus in different laboratories may produce differing phenotypes as a result of it encoding a different repertoire of genes, such as the multiple genetic variants of AD169 and Towne (7). An example of an incorrect conclusion resulting from use of a passaged strain is the initial description of HCMV-induced upregulation of CD58 (11), when wildtype viruses actually impair expression (12).

Therefore, to understand pathogenicity *in vivo*, the use of strains encoding the complete repertoire of HCMV genes is desirable. We and others have endeavored to meet this need by generating infectious bacterial artificial chromosomes (BACs) containing cloned HCMV genomes (3). BACs enable a HCMV genome to be stably maintained in *E. coli*, without the risk of selection of mutants as occurs during mammalian cell passage. Virus can then be repeatedly re-derived from a consistent genetic starting point. Furthermore, techniques such as recombineering or *en-passant* mutagenesis can be used to rapidly modify the cloned genome (13, 14). This has resulted in the generation of numerous BAC cloned HCMV strains. Unfortunately, to accommodate BAC sequences, many of the initially created BACs contained deletions in the US2-6 region (3), within which there are genes that downregulate HLA-I from the cell surface, as well as genes that act as hubs for degradation of multiple additional host proteins. The higher HLA-I levels affect readouts of NK and T-cell activation, while the loss of US2 affects cell migration, adhesion, and NK cell interactions (15). Furthermore, when these BACs are transfected to recover virus, additional mutations can occur around the BAC cassette, and in the U_L_/b′ region (16). Taken together, these issues mean that NK assays using viruses derived from these early BACs need to be interpreted with caution.

More recently, versions of some of these BACs have been produced in which the deleted sequences from the US region have been re-inserted, and the BAC cassette excised to accommodate the additional material (17–19). These strains provide a convenient reagent, nevertheless, results from them still need to be interpreted with caution as most lack UL141, a major NK regulator, and carry mutations in two genes (RL13 and UL128), that significantly affect tropism and virus growth properties (20). Furthermore, the original clinical material is not available, making it impossible to know whether additional *in vitro* acquired mutations are present. Such mutations can be as subtle as a change in a splice site (20), or an amino acid alteration (21), yet can have significant impacts on virus:host interactions (3).

Our BAC of choice currently is therefore Merlin, which was isolated from the urine of a congenitally infected child, and cloned into a BAC as a full-length genome (9). BAC sequences were also excised from derived viruses, ensuring that they do not drive the selection of mutations (16). Comparison of the BAC sequence to the sequence of the virus found in the original clinical sample revealed *in vitro* acquired mutations in two genes, UL128 and RL13, which were then repaired. This generated the only BAC to date that contains a full length HCMV genome that matches the original clinical sample, and encodes the complete repertoire of wildtype HCMV genes (9). Further work enabled Merlin containing a full-length genome to be passaged *in vitro* without selection of mutants (16). Interestingly, unlike passaged HCMV strains, viruses derived from this BAC (i.e., containing RL13 and UL128) do not release substantial amounts of infectious cell-free virus, instead spreading predominantly by the cell-cell route. This is consistent with observations that clinical viruses do not release high levels of cell free virus *in vivo* or *in vitro* (6, 9, 22). This distinction is important for understanding HCMV's *in vivo* interactions with the immune system, as firstly, cell-cell spread is more resistant to elements of innate and intrinsic immunity; secondly, cell-cell spread is more resistant to neutralizing antibodies than cell-free spread; and finally, cell-cell spread enables highly efficient transfer of wildtype virus into a wide range of cell types, including macrophages, dendritic cells, endothelial cells and epithelial cells that are infected by HCMV *in vivo* (22).

**Figure 1:**
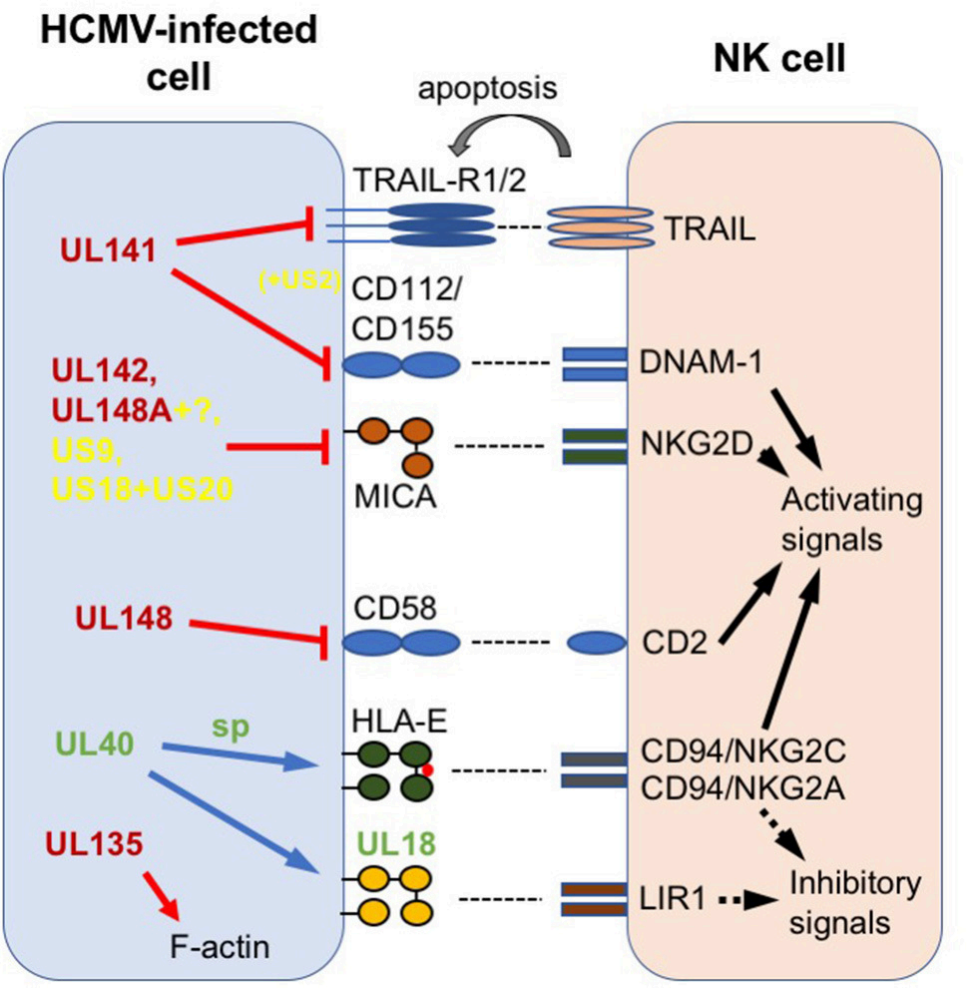
Summary of HCMV-encoded NK modulators discussed in this review. Host proteins are labeled in black; HCMV proteins in red (found in U_L_/b′ region), green (variable outside U_L_/b′) or yellow (conserved outside U_L_/b′); sp = signal peptide. Arrows and lines represent actions. Solid black arrow = intracellular NK activation signal; dotted black arrow = intracellular NK inhibition signal; gray arrow = extracellular signal to target; red line = impairs surface expression; red arrow = disrupts intracellular expression; blue arrow = increases surface expression.

These reagents have allowed us and others to study gene function in the context of HCMV infection on a stable, close-to-wildtype, genetic background. However, the loss of functions from passaged “laboratory” strains still serves a useful role, in that it can focus research on genetic regions that contain proteins encoding accessory functions that are selected for *in vivo*, such as those involved in responding to the immune system. In this context, studies comparing the host response to strains containing an intact U_L_/b′ region with those lacking this region showed significant differences, correlating loss of U_L_/b′ with reduced pathogenicity following *in vivo* challenge (23, 24), as well as greatly enhanced sensitivity to NK cell killing (25–27). Subsequent work has identified a range of immunomodulatory functions in the U_L_/b′ region, with this review detailing those that specifically target NK cells, and discussing the novel aspects of NK biology that study of the U_L_/b′ region has revealed. In addition to alterations in NK modulators occurring due to selection *in vitro*, NK modulatory genes outside the U_L_/b′ region have also undergone selection *in vivo* to give rise to genetic variation. We therefore finish with a discussion of recent research into this selection, highlighting how important HCMV:host interactions are defined by their impact on the phenotype of NK cells following exposure to HCMV. The different HCMV-encoded NK evasins described in this review are summarized in Figure 1.

### NK cell modulators lost from HCMV in cell culture

#### Targeting of ligands for DNAM-1 and TRAIL-Rs by UL141

The first HCMV U_L_/b′ gene to be designated an NK cell evasion was UL141, and occurred prior to the development of BAC technology. Use of a recombinant HCMV consisting of Towne and the U_L_/b′ region from Toledo revealed that transferring the U_L_/b′ region conferred resistance to NK cell killing. UL141 was then identified as one of the genes responsible for this phenotype, by screening individual Toledo U_L_/b′ genes, ectopically expressed from replication-deficient adenoviruses (rAds). The cellular protein targeted by UL141 to achieve this effect was identified through an extensive screen of known NK cell activating ligands on the surface of rAdUL141-infected cells. Surface expression of CD155 (poliovirus receptor/nectin-like molecule 5), a protein normally involved in formation of stromal cell-cell junctions (28) and an activating ligand for DNAM-1 (CD226) and CD96 on NK cells (29, 30), is impaired by its binding and sequestration inside the endoplasmic reticulum (ER) by UL141. Demonstration of UL141 function in the context of infection was achieved through subcloning a HCMVΔUL141 strain from TB40/E (27).

CD112 (nectin-2), another ligand for DNAM-1 (29), is also downregulated upon HCMV infection and rescued by deletion of UL141 from Merlin, albeit by a distinct mechanism (31). In this case, UL141 acts in concert with a second HCMV gene outside the U_L_/b′ region. Plasma membrane profiling of cells infected with HCMVs lacking each of the HLA-I downregulating genes (US2, US3, US6, and US11) revealed that UL141 co-operates with US2 to degrade CD112 in a TRC8-dependent manner, the same mechanism by which US2 induces HLA-I heavy chain proteasomal degradation (15).

Two further host proteins impacting on NK function have been identified that are modified by UL141. TNF-related apoptosis inducing ligand receptors 1 and 2 (TRAIL-R1 and R2) are able to transmit apoptotic signals to the cell through adapter molecules leading to caspase activation following ligation by their ligand, TRAIL/Apo2L (32). UL141 is responsible for downregulation of both TRAIL-R1 and R2 during HCMV infection, also through retention in the ER (33, 34). Apoptosis assays demonstrated that overexpression of UL141 through rAds protected, while cells infected with HCMVΔUL141 on a strain FIX background were more susceptible, to TRAIL-dependent NK cell killing (34). Thus, UL141 is remarkable in that it prevents NK cell mediated cytotoxicity by targeting at least 4 different proteins using two independent mechanisms of intracellular retention and proteasomal degradation. Three separate background strains (TB40/E, Merlin and FIX) were used to generate HCMVΔUL141 viruses in these studies.

#### Targeting of MICA by UL142 and UL148A

Interest in UL142 was driven by the prediction that it had homology to HLA-I following analysis of UL142 sequences from high and low passage HCMV strains with ORF prediction tools. Cells which ectopically expressed UL142 were more resistant to NK cell mediated killing (35). This was achieved through downregulation and sequestration into the cis-Golgi of MHC-I related protein A (MICA) (36, 37), one of the ligands for the activating receptor Natural-killer group 2, member D (NKG2D) found on T and NK cells. UL142 is polymorphic in clinical HCMV isolates (36), and it was proposed that this is to accommodate for diversity in MIC alleles. Indeed, MICA^*^008, which is the most common MICA allele, and lacks a cytoplasmic tail due to a premature stop codon, is resistant to UL142 mediated downregulation (36, 38). Instead, US9 specifically targets MICA^*^008 for proteasomal degradation, without affecting other MICA or HLA-I-like molecules. Here HCMVΔUS9 was generated on an AD169VarL background missing UL140-UL144 rather than the whole U_L_/b′ region (39). Downregulation of MICA has also been mapped to UL148A (40), as well as genes in the US18-22 region of the Merlin genome (41). UL148A was identified using HCMV knock-out viruses on an AD169VarL background, and requires an as-yet unidentified additional HCMV gene to function (40). Interestingly, proteomic analysis comparing the impact of AD169 (lacking UL148A and the rest of the U_L_/b′ region) and Merlin infection on fibroblasts did not show differential effects on MICA (42), suggesting the potential for strain-specific interactions between this region and the host. Deletion of US18 and US20 individually and together indicated that these two genes act synergistically to inhibit MICA expression (41). UL148A, US18, and US20 all traffic MICA to the lysosome for degradation. Multiplexed proteomic analysis of the same gene region revealed that US18 and US20 also impair NK recognition by targeting the NKp30 ligand B7-H6, underscoring the ability of viral immune-evasions to modulate multiple cellular pathways simultaneously (43, 44).

#### Disruption of the actin cytoskeleton by UL135

A systematic screen of genes in the U_L_/b′ region using ectopic expression by rAds showed that UL135 also impaired the degranulation of both NK and T cells (45). Compared with Merlin, cells infected with MerlinΔUL135 were less rounded when assessed by microscopy, implying that UL135 had an important role in the characteristic cytopathic effect induced by HCMV. In support of this, MerlinΔUL135-infected cells did not exhibit loss of F-actin from the center of the cell that is normally observed following HCMV infection. Using yeast-2-hybrid and Stable Isotope Labeling with Amino acids in Cell culture combined with immunoprecipitation (SILAC-IP), UL135 was found to interact directly with AB1/AB2, and via these, relocated the entire WAVE2 regulatory complex (WRC; including ABI1, ABI2, NAP1, CYFIP1, WAVE2). Importantly, the WRC is responsible for regulating the actin nucleator Arp2/3 (46). The interaction with the WRC was critical for both the UL135-mediated cytopathic effect, and immune evasion, when assessed by NK cell degranulation and adhesion. Furthermore, imaging revealed a correlation between the presence of actin fibers in the target cell, with the structure of the immune synapse formed with NK cells. When F-actin fibers in the target cell were disrupted by UL135 expression, the ability of the NK cell to form an immune synapse was impaired. Thus it appears that in addition to well recognized structural requirements in the effector cell, physical structures in the target cell are also required for the formation of effective immunological synapses, and this requirement is targeted by HCMV to mediate immune-evasion (45).

#### Suppression of co-stimulation by UL148

CD2 is an important co-stimulatory molecule found on the surface of T and NK cells and binds to CD58 (LFA-3) on the surface of antigen presenting cells (47). Recent work has identified the role of CD58:CD2 signaling as important for adaptive NK cell activity, regulating cell adhesion and the formation of the immunological synapse (48). Early research showed that CD58 was upregulated on the surface of HCMV-infected cells following AD169 infection (11), and it was suggested that this was responsible for aiding the adhesion of CD2^+^ lymphocytes to HCMV-infected cells. Later research showed that the difference in CD58 expression on HCMV-infected cells correlated with the amount of NK mediated killing (49). This hypothesis was further supported recently, when it was shown that blocking CD58 on the surface of AD169-infected MRC-5 fibroblasts resulted in reduced IFNγ and TNF production by NK cells (50).

However, this contrasted with data demonstrating downregulation of CD58 on the surface of mature and immature dendritic cells infected with clinical strains of HCMV (51, 52). The basis of these contradictory results was uncovered when we showed that HCMV infection with Merlin also led to a decrease in CD58 on the surface of fibroblasts (44, 53), as a result of UL148-mediated sequestration in the ER (12). Functionally, this reduced both CD8^+^ T and NK cell activation. The effect of UL148 was greatest on NK cell activation in the context of antibody dependent cellular cytotoxicity (ADCC), which is consistent with reports that the CD2:CD58 pathway contributes significantly to this process (54). Perhaps even more significantly for the host, ADCC was detected against Merlin-infected cells regardless of a full complement of NK evasion genes. The CD57^+^ population of adaptive NK cells, increases in which are associated with previous HCMV infection (55), showed the most sensitivity to inhibition induced by UL148 (12).

### Other genes in U_L_/B′ with the potential to modulate NK cells

Published systematic screen of genes within the U_L_/b′ region were completed with a single cytotoxic readout using expression of individual genes, and did not identify any other obvious NK evasions (45). There is, however, great complexity in HCMV's interaction with the immune system, with examples of single HCMV genes acting on multiple host proteins (e.g., UL141 targeting CD155 and TRAIL-Rs) or multiple HCMV genes synergizing to act on a single host protein (e.g., UL141 and US2 against CD112). The latter function would not have been found using a single gene expression system of screening, requiring extended analysis with HCMV knockouts instead. Furthermore, altering readouts may reveal otherwise hidden functions such as those observed with UL148's specific impact on ADCC (12). Thus, there remains considerable potential for additional NK immune modulators to be identified within the U_L_/b′ region. A number of these are genes that exhibit high intrastrain variability, in particular UL144, UL146, and UL147 (56), although no functional differences have yet been reported to be associated with these variants.

UL144 encodes an ortholog for the herpes virus entry mediator (HVEM) (57), a member of the TNFR superfamily (TNFRSF) with multiple immunomodulatory ligands including LIGHT, CD160, B- and T-lymphocyte attenuator (BTLA) (58) and lymphotoxin (LT). Treatment of HCMV-infected cells with LT and LIGHT results in reduced spread of HCMV (59). As receptors for HVEM are found on T and NK cells, it was predicted that UL144 would affect both these effector subsets. Significantly reduced proliferation of CD4^+^ T-cells has been reported in the presence of plate bound UL144 (60). This was attributed to the binding of UL144 to BTLA, a co-inhibitory receptor. Unlike HVEM, UL144 cannot bind to CD160, which normally provides a costimulatory signal for NK-cells promoting cytolytic activity. Thus, UL144 has been proposed to provide a specific inhibitory signal via BTLA counterbalancing HVEM-induced activation signals through CD160, but to date, actual inhibitory function for UL144 on NK cells has not been demonstrated (61). Whilst UL144 is considered variable, differences in UL144 genotype have not been reported to carry prognostic value in infected fetuses or predict severity of disease (62, 63).

UL144 may also have additional effects as it causes constitutive NF-kB activation in isolation via recruitment of TRAF-6 (64). This can result in expression of CCL22, a chemotactic factor, which may affect migration of CCR4 expressing NK cells (65). The microRNA-UL148D has also been shown in a lytic infection to target another chemokine RANTES (regulated on activation, normal T-cell expressed and secreted). The ligand for RANTES, CCR5, can be detected on CD16^−^ NK cells, which migrate upon RANTES treatment (66). Therefore, the prevention of RANTES production may potentially affect the migration of NK cells toward HCMV infected cells. UL146 and UL147 encode two additional viral chemokine homologs vCXCL1 and vCXCL2 respectively (58). UL146 increases NK cell migration (67).

### Natural variants of other HCMV-encoded NK modulators

Beyond *in vitro* acquired genetic variation, a number of HCMV-encoded NK modulatory genes are naturally variable, likely representing *in vivo* selection of virus genotypes by the immune system. UL40 was the first HCMV-encoded NK evasion shown to act during an active HCMV infection using a HCMVΔUL40 on an AD169 background (26). This was achieved by TAP-independent transfer of a peptide in the UL40 leader sequence to bind and upregulate HLA-E, which then acts as a ligand for the inhibitory CD94^+^NKG2A^+^ complex on NK cells (68, 69). However, HLA-E also binds the activating CD94^+^NKG2C^+^ complex with lower affinity (70), and silencing of HLA-E using shRNAs inhibits the *in vitro* expansion of adaptive NKG2C^+^ NK cells driven by AD169-infected fibroblasts (71). UL40 itself is hypervariable in HCMV strains, exhibiting mutations that can abrogate UL40 expression altogether, or alter its HLA-E binding peptide. The latter have now been shown to differentially inhibit killing of HLA-E expressing RMA-S cells by NKG2A^+^ NK clones (72) and drive NKG2C^+^ NK proliferation stimulated by the same cells (19). Hammer and colleagues also elegantly demonstrated differential activation of NKG2C^+^ NK cells by endothelial cells infected with repaired TB40 containing the various UL40 mutants, highlighting one underlying mechanism driving the activation and expansion of adaptive NK cells following HCMV infection. This cannot, however, be the only mechanism as NK expansions are also observed in HCMV seropositive NKG2C^null^ individuals (73).

There is also natural variation in UL18, the HLA-I homolog which binds to the inhibitory leukocyte immunoglobulin like receptor (LIR1, LILBR1) (74, 75). These variants exhibit altered capacities to bind LIR1 (76, 77) and differentiate a HCMV strains' susceptibility to LIR1^+^ NK cell control in viral spread assays (78). UL18 can vary by up to 20 amino acids (76), and this occurs outside of the peptide binding groove (79). The affinity of LIR1 for UL18, but not its host ligand HLA-G, is affected by genetic variation in the LIR1 gene (80). The differences in LIR1 genotype correlate with control of HCMV disease in renal transplant patients and altered NK-cell activation (81). Thus, UL18 is an example whereby a small change in the binding affinity between a viral and host protein can be mapped to specific SNPs, which can predict disease outcome following transplantation. To add further complexity, UL40 can upregulate the expression of UL18, even when truncation cleaves the HLA-E binding peptide and disrupts upregulation of HLA-E suggesting distinct functions (82). The impact of UL40 variants on UL18 expression, and therefore on LIR-1^+^ NK cells, remains unknown.

In general, however, the majority of HCMV-encoded NK modulators are not considered particularly variable. A summary of all of those with an established direct impact on NK activation, and an indication of their degree of genetic variability and whether they are found in the U_L_/b′ region is provided in Table 1. It is perhaps of note that where variability does occur, the affected proteins interact with host systems that are in turn very variable: UL142 targets MICA that is highly polymorphic; UL40 provides peptides that bind HLA-E; while UL18's ligand LIR1 interacts with the most polymorphic human gene family of all, HLA-I. Furthermore, our current knowledge suggests that MICA alleles are targeted by at least 5 HCMV genes (UL142, UL148A, US9, US18, and US20), while the less polymorphic MICB and ULBPs only by two (UL16, miRNA-UL112). Thus, HCMV may have to dedicate more of its genome to tackling highly polymorphic immune selection. Only further research will determine how significant these associations are.

**Table 1:** Summary of established modulators of NK activation, their genetic variability and presence in U_L_/b′ region.

**Gene**	**Function**	**Variability^a^**	**U_L_/b^′^**	**References**
RL11	Binds Fcγ receptors, reducing NK cell mediated ADCC	Low	No	83
UL16	Sequesters MICB and ULBPs	Low	No	(84, 85)
UL18	MHC-1 homolog which binds LIR1	Low^b^	No	(74, 75)
UL40	UL40 signal peptide upregulates HLA-E. Variation alters responsiveness of NKG2C^+^ NK cells	Low^c^	No	(68, 19)
UL83	Binds and prevents activation of NKp30 on NK cells	Low	No	(86)
miRNA-UL112	Binds to MICB RNA, reducing MICB surface expression	Low	No	(87)
UL119-UL118	Binds Fcγ receptors, reducing NK cell mediated ADCC	Low	No	(83)
UL148A	Downregulates MICA in conjunction with as yet unidentified HCMV gene	Low	Yes	(40)
UL148	Impairs CD58 (LFA-3) surface expression. Inhibition of CD8^+^ T as well as NK cell mediated ADCC	Low	Yes	(12)
UL142	HLA-I homolog that downregulates MICA (not the MICA*008 allele)	Medium	Yes	(35, 36, 37)
UL141	Downregulates CD155, CD112, TRAIL-R1, and TRAIL-R2. Requires US2 to target CD112	Low	Yes	(27, 31, 34)
UL135	Remodels actin cytoskeleton. Inhibition of CD8^+^ T as well as NK cells	Low	Yes	(45)
US9	Sequesters MICA*008	Low	No	(39)
US18 and US20	Targets MICA and B7-H6 for degradation	Low	No	(41, 44)

a *Level of genetic variation as described in Sijmons et al. 56*.

b *Genetic variation is high in the signal peptide domain of UL40 72*.

c *Variation occurs in the non-peptide binding domain of UL18 79*.

## Concluding remarks

Investigating regions of genetic instability has aided us in identifying and focusing research on several of HCMV's impressive arsenal of NK evasion functions. Caution is, however, required when interpreting data from these passaged viruses as they generally do not fully represent the clinical agent in respect to biological properties, process of infection, tropism and immune evasion. Unfortunately, the use of clinical isolates is also extremely problematic because they produce very low titers of infectious virus, they can exhibit natural genetic deletions, and mutants are selected very rapidly following *in vitro* isolation. The production of BAC-cloned viruses containing verified wildtype genomes offers a solution, however even with these, the presence of natural variants that alter NK responses means that ideally multiple strains should be compared. Despite these difficulties, the extra effort to study HCMV in settings as close as possible to those found *in vivo* will be worthwhile as it is likely to shed light on the underlying reasons why infection induces such unusual NK and T-cell expansions, which will in turn inform on the potential use of HCMV as an unique vaccine vector. Uncovering individual functions of novel HCMV-encoded immune modulators will undoubtedly continue to further our knowledge of HCMV virulence factors and our understanding of basic NK cell biology, but more global approaches that take into account selection-driven genetic variation and multiple gene interactions will be needed to fully understand how we can manipulate HCMV for our own ends.

## Author contributions

MP wrote the first draft of the manuscript. V-MV, SF, CF, RS, and EW wrote additional sections. All authors contributed to manuscript revision, read and approved the submitted version.

### Conflict of interest statement

The authors declare that the research was conducted in the absence of any commercial or financial relationships that could be construed as a potential conflict of interest.
